# Pelvic Floor Dysfunction among Reproductive-Age Women in Israel: Prevalence and Attitudes—A Cross-Sectional Study

**DOI:** 10.3390/healthcare12030390

**Published:** 2024-02-02

**Authors:** Tehila Fisher-Yosef, Dina Lidsky Sachs, Shiri Sacha Edel, Hanan Nammouz, Abd Ellatif Zoabi, Limor Adler

**Affiliations:** 1 Health Division, Maccabi Healthcare Services, Tel Aviv 6812509, Israel; 2 Faculty of Medicine, Tel Aviv University, Tel Aviv 6997801, Israel; 3 The Azrieli Faculty of Medicine, Bar Ilan University, Zefat 1311502, Israel

**Keywords:** pelvic floor dysfunction, women, parous, reproductive-age women, attitudes, prevalence

## Abstract

**Objectives:** Our study aimed to investigate the prevalence of female pelvic floor dysfunction (PFD) in Israeli women who experienced vaginal delivery and are in their reproductive years (premenopausal), as well as to understand their attitudes and health-seeking behavior and barriers towards treating this problem. **Methods:** In this cross-sectional study, we conducted a questionnaire-based Internet survey. The surveys were sent to Israeli women in their fertile years (18–50 years old). We asked the women about their PFD symptoms, attitudes, and help-seeking behaviors. We used two validated questionnaires, including the USIQ and the PFDI-20. The combined questionnaire was submitted in both Hebrew and Arabic. We assessed the prevalence of PFD symptoms in the study population. Symptomatic women were asked about their help-seeking behaviors and their beliefs, desires, and barriers regarding the clinical management of symptoms. **Results:** Between July and September 2020, 524 women completed the questionnaire (response rate 44%). In total, 95% reported at least one symptom (mostly urinary-related) at any grade of severeness in at least one category, and 66.8% suffered from at least one moderate to severe symptom in at least one category. Most women (93.7%) reported that they wanted to be asked and offered voluntary information about PFD from physicians and nurses; however, only 16.6% reported receiving such information. Barriers to seeking treatment were mainly related to low awareness. The study’s main limitation was selection bias due to the questionnaire’s design. **Conclusions:** These findings show the importance of raising awareness of the different therapeutic solutions to PFD symptoms and designing more available services for this common problem.

## 1. Introduction

Female pelvic floor dysfunction (PFD) is a term applied to a wide variety of clinical conditions, including urinary incontinence (stress, urge, and mixed), fecal and flatus incontinence, pelvic organ prolapse (POP), constipation, sexual dysfunction, and several chronic pelvic pain syndromes [1,2,3]. These conditions may cause significant suffering and impair the quality of life for many women, affecting their mental and sexual health as well as their social and physical activities [4,5,6]. The disorder is considered multifactorial, with vaginal parity being one of the leading risk factors for developing it [2,3,7,8], with a positive correlation to the number of vaginal births [4,7,8]. Assisted vaginal births (i.e., forceps and vacuum deliveries) were found to be contributing factors [2,5], as well as an extended second phase of labor (over 1 h), history of 3rd- or 4th-degree perineal tears, obesity, older age, positive family history of PFD, and smoking [2,6,7,8,9].

Estimating the prevalence of PFD is important for several reasons, including assessing the public health burden of these conditions as well as implementing healthcare strategies. Different studies have examined the prevalence of these conditions, usually using voluntary questionnaires [2,5,10,11,12,13,14,15,16,17,18,19]. Some studies have focused on urinary symptoms alone, some on the postpartum period, and some on the postmenopausal period. Correspondingly, there is a wide variation in the prevalence of PFD when comparing these studies and others, and any figure between 1.9 and 67% can be found. However, it is, without a doubt, not a rare condition. Nevertheless, the data on women in their reproductive years beyond the peripartum period are scarce. 

PFD is a largely treatable problem. Pelvic floor muscle training (with or without biofeedback) has been shown to improve urinary incontinence, pain, and quality of life [20,21,22,23]; pessary surgery (implantation of sub-urethral tension-free slings, symmetric lateral levator myography colposuspension) is also an available treatment [23,24,25], as are botulinum toxin injections in selected cases [23]. Without early intervention, PFD may deteriorate and require more invasive and costly treatments. In a Cochrane meta-analysis by Dumoulin et al. involving a total of 165 patients, there was a cure rate of 56.1% in those who performed pelvic floor exercises and only 6% in those who did not (relative risk [RR] 8.38, 95% CI 3.68–19.07) [26]. The treated groups had significantly better outcomes concerning their quality of life, satisfaction with treatment, and need for further treatment compared with the control groups. Leaving PFD untreated could also be very costly: One study estimated the costs of ambulatory treatment for PFD in the United States to be as high as USD 300 million between 2005 and 2006 [27]. Left untreated, PFD has negative effects on women’s health and quality of life, affecting their physical and mental health and sometimes even leading to social isolation, anxiety, and depression [28,29].

Despite the nature of PFD as a preventable and treatable condition affecting everyday life, most women avoid discussing this issue when encountering healthcare professionals [12,18,30,31]. The reasons for this are various: Some women have the perception that PFD is a normal part of aging, some expect it to wane eventually without intervention, some are too embarrassed to raise the issue, and some see it as an inevitable and untreatable adverse effect of parity, i.e., are unaware that treatment is available [18,32]. As a result, many women are left untreated or wait many years before seeking medical help [12,32]. 

Our study aimed to investigate the prevalence of PFD in Israeli parous women in their reproductive years and to investigate their needs, attitudes, barriers, and health-seeking behavior surrounding this problem. In addition, we wanted to evaluate which social-demographic and medical factors were associated with PFD and moderate-to-severe PFD. This would ideally assist us in understanding the key obstacles associated with addressing this medical condition and enhancing the quality of life for women.

## 2. Methods and Materials

### 2.1. Setting and Study Design

In this cross-sectional study, we conducted a questionnaire-based internet survey using PharmaQuest Ltd. (Ramat-Gan, Israel) company’s platform. The company has an advanced, fully secured online system that enables questionnaire distribution and de-identified data collection that fully complies with EphMRA, ESOMAR, and ethical codes of conduct. It is a private independent research company and is not affiliated with any product.

The company distributes advertisements and invitations to join its databases on various websites such as Google and Facebook. Every internet-accessible citizen can be exposed to these invitations and links and can join the database, which is free and on a voluntary basis. Afterwards, a comparison is conducted between the demographic details of registered individuals and the overall demographic profile of Israeli citizens, as provided by the Central Bureau of Statistics in Israel, to generate a representative sample. The sample includes 100,000 people representing Israeli society. Registered individuals respond to population surveys and accumulate points with which they can purchase vouchers.

The company can activate filters when distributing questionnaires, such as selecting specific age groups or a particular gender. 

The e-mail provided details about the study and invited the women to participate in an anonymous survey. Those who satisfied the age and parity inclusion criteria, determined by initial filtering questions, were allowed to proceed to the full questionnaire.

Within the survey, we inquired about the PFD symptoms, attitudes, and behaviors related to seeking help. The funding for these services was sourced from Marom, a research program catering to physicians and residents affiliated with Maccabi Healthcare Services.

### 2.2. Study Population

Inclusion criteria: women who experienced at least one vaginal delivery and are in their fertile years (18–50 years old).

### 2.3. The Questionnaire

We used two validated and well-established questionnaires for detecting PFD and assessing its effect on quality of life: the Pelvic Floor Distress Inventory (PFDI-20) [33,34,35] and the Urgency Severity and Impact Questionnaire (USIQ) [13,35]. The PFDI-20 questionnaire is a 20-item questionnaire divided into 6 items evaluating pelvic organ prolapse distress, eight items evaluating colorectal anal distress, and six items evaluating urinary distress. The USIQ focuses on urge urinary incontinence and has two parts: symptom severity and related quality of life. It includes 14 questions. Both questionnaires were previously validated in Hebrew [34,35]. After receiving permission from the original authors, we translated the questionnaires into Arabic using reverse translation and validation tools. Three native Arabic speaker doctors reviewed the questionnaire before distribution. Then, we designed a complete questionnaire that included four sections (see the full version in Supplementary Materials):

(a) Filtering questions for inclusion criteria (age, previous vaginal delivery)—2 questions;
(b) PFDI and USIQ questions for identifying symptomatic women—34 questions in total. These questions provided information on the prevalence of PFD in the study population;
(c) Beliefs, attitude, barriers, and treatment-seeking behaviors regarding PFD—9 questions. These questions explored symptomatic women’s beliefs about their symptoms, willingness to seek and explore different medical solutions, and ability to adhere to recommended treatment methods;
(d) Medical, social, and demographic questions—18 questions. These included: age, number of births (both vaginal and cesarean), years since last delivery, types of vaginal deliveries (assisted or not), history of episiotomy, weight, height, smoking status, history of hormonal medication—both consumption and duration, ethnicity, education, marital status, and socio-economic status (based on income and residence).

The questionnaire was submitted in both Hebrew and Arabic. An English translation is available in the Supplementary Materials. 

We divided the PFDI and USIQ answers into five categories: (1) bladder symptoms—mainly stress incontinence; (2) bladder symptoms—mainly overactive bladder/urge incontinence; (3) colorectal symptoms—mainly obstructive, i.e., constipation; (4) colorectal symptoms involving mainly incontinence—flatus and/or fecal; and (5) pelvic pain or discomfort and pelvic organ prolapse. 

Each category was given a severity scale corresponding to the questionnaires we used (ranging from 0, as in never having these symptoms, to 5, as in having symptoms and finding them considerably bothersome). A score above 0 in each category was sufficient to define the patient as symptomatic, and a score of 3 and above (i.e., “yes, and it bothers me to a minor/moderate/great extent”) defined a patient with moderate to severe symptoms. 

### 2.4. Statistical Analysis

We used descriptive statistics to characterize the variables, including mean and standard deviation (SD) for continuous variables and counts and percentages for categorical variables. For the univariate comparison of continuous variables, we used the Student *t*-test for standard distribution variables and Mann–Whitney for non-normal distribution variables. For categorical variables, we used the chi-square test. We performed a multivariate analysis with logistic regression to test which factors were associated with PFD and moderate to severe PFD. In this regression, we inserted all sociodemographic and medical variables to test which affected the presentation of symptoms; these variables included age, number of vaginal deliveries, time since the last delivery, perineal stitching, assisted delivery (forceps/vacuum), socio-economic status, ethnic background (Arab, Orthodox Jewish, all others), BMI, and smoking. A *p* value < 0.05 was considered significant. All analyses were performed using SPSS Statistics version 27 (SPSS Inc., Chicago, IL, USA). 

### 2.5. Ethical Considerations

The institutional review board of Assuta Health Care ASMC-0110-18 approved the study. Study participation was voluntary and anonymous. Consent to participate was granted by submission of a completed questionnaire.

## 3. Results

### 3.1. Participants 

From July to September 2020, A total of 25,000 personal e-mail invitations were sent to all women under the age of 50 in the database. A total of 9907 women (4633 native Hebrew speakers and 5274 native Arabic speakers) accessed the questionnaire. This confirmed that they had seen the survey. Participants who answered the full survey were granted 20 NIS worth of vouchers. 

**Figure 1:**
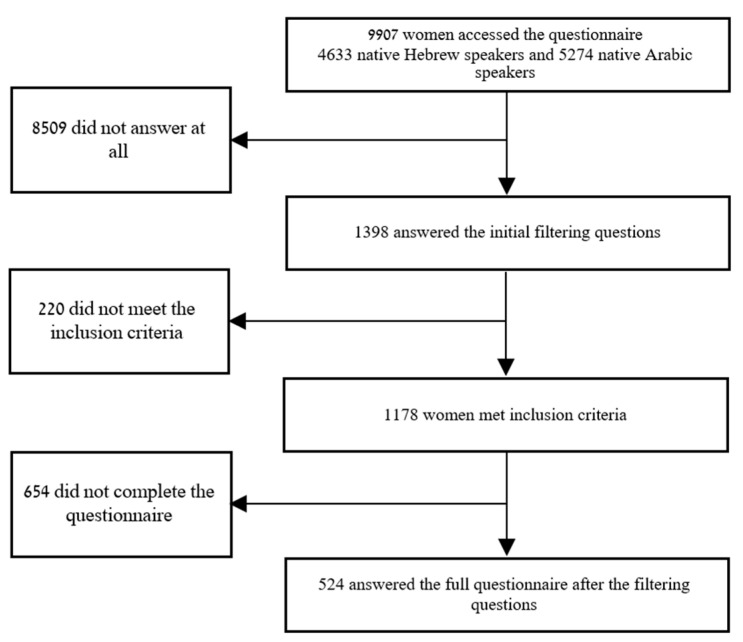
Flowchart.

The invitation to participate in the survey was emailed on six different occasions over these months (approximately once every two weeks). One thousand three hundred ninety-eight women answered the initial filtering questions. Out of the participants who fulfilled the inclusion criteria based on the initial filtering questions (i.e., being between the ages of 18–50 and having undergone at least one vaginal delivery), a total of 1178 women were identified. Among these, 524 completed the full questionnaire. They were considered responders, and 654 did not complete the rest of the questionnaire and were considered non-responders, resulting in a response rate of 44% for eligible participants (Figure 1). 

### 3.2. Descriptive Data

Among the participants who completed the questionnaire, 80.5% (422) belonged to the Jewish sector, and 19.5% (102) represented the Arab sector. The average age was 35.9, with a standard deviation of 7.6 years. Most participants had an academic background, comprising 61.8% (324 individuals).

The average number of vaginal deliveries was 5.1, with a median of 3. In terms of delivery methods, 21.6% (113) had undergone both vaginal and cesarean deliveries, while 78.4% (411) had undergone exclusively vaginal deliveries. Furthermore, 17.6% of the women (92) had a history of at least one instrumental delivery (forceps or vacuum). Most women (72.1%, 378) required perineal stitches in at least one delivery. At the time of the questionnaire, 38.1% of the women (200) were at least five years postpartum, 39.7% (208) were between one and five years postpartum, and 22.2% (116) were less than a year postpartum. A positive smoking status was reported by 11.6% (61) of the respondents. 

Stress urinary incontinence: A total of 370 participants (70.6%) reported having at least one symptom, and 193 (36.8%) reported moderate to severe symptoms. 

Overactive bladder/urge urinary incontinence: A total of 394 participants (75.2%) reported having at least one symptom, and 215 (41%) reported moderate to severe symptoms.

Obstructive colorectal symptoms: A total of 331 participants (63.2%) reported having at least one symptom, and 150 (28.6%) reported moderate to severe symptoms.

Colorectal incontinence (gas and/or feces): A total of 325 participants (62%) reported having at least one symptom, and 123 (23.5%) reported moderate to severe symptoms.

Pelvic pain or discomfort and prolapse: A total of 433 participants (82.6%) reported having at least one symptom, and 242 (46.2%) reported moderate to severe symptoms.

**Figure 2:**
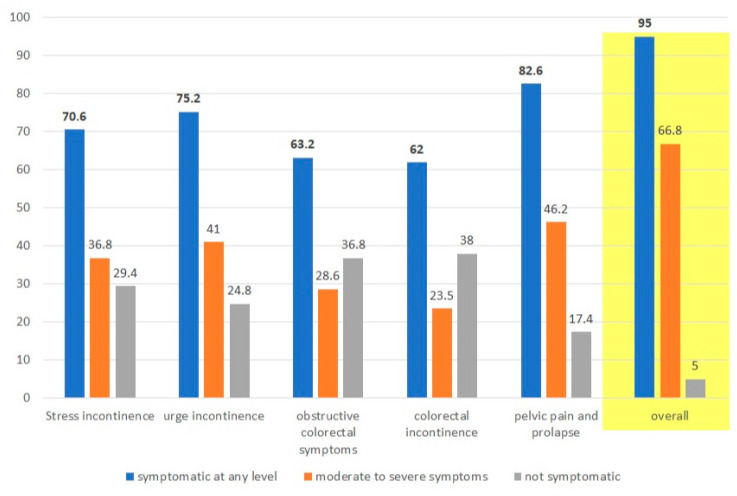
PFD prevalence in the study population.

Overall, 498 participants (95%) suffered from at least one symptom at any grade in at least one category, and 350 participants (66.8%) suffered from at least one moderate to severe symptom in at least one category (Figure 2). The differences in women with and without moderate to severe symptoms are outlined in Table 1. 

The most common complaints were urinary-related (stress incontinence or overactive bladder). A total of 447 participants (85.3%) suffered from at least one urinary symptom in any grade, and 259 participants (49.4%) suffered from at least one moderate to severe symptom. 

### 3.3. Multivariate Analysis

In this study, there was no effect of age, number of vaginal deliveries, time since the last delivery, perineal stitching, assisted delivery (forceps/vacuum), or socio-economic status on the chance of suffering from PFD symptoms. Nevertheless, women in the Arab sector tended to suffer more from pelvic pain or pelvic organ prolapse (OR 2.046, CI [1.004–4.173], *p* = 0.049), obstructive colorectal symptoms (OR 2.017, CI [1.185–3.432], *p* = 0.01), and moderate to severe colorectal incontinence (OR 1.730, CI [1.035–2.892], *p* = 0.036). A BMI greater than 25 was found to be a risk factor for suffering from fecal incontinence (OR 1.043, CI [1.004–1.084], *p* = 0.029), pelvic pain, or organ prolapse (OR 1.045, CI [1.007–1.084], *p* = 0.019), as well as moderate to severe urinary stress incontinence (OR 1.085, CI [1.044–1.126], *p* < 0.001)

Smoking was found to increase the risk of colorectal obstruction symptoms by almost twofold (OR 1.995, CI [1.033–3.854], *p* = 0.04), an effect that was also found in stress urinary incontinence (OR 2.362, CI [1.137–4.907], *p* = 0.021), and moderate to severe overactive bladder symptoms (OR 2.062, CI [1.161–3.661], *p* = 0.014).

### 3.4. Quality of Life Assessment

In the questionnaire, women were asked to grade the influence of urinary urge incontinence on their daily lives on a scale of 0-4 according to the USIQ questionnaire (“How much does it affect your ability to…”), where zero corresponded to “Not at all”, 1 to “Somewhat”, 2 to “Moderately”, 3 to ”quite a bit”, and 4 to “Very much”. Urinary urge incontinence was found to influence many daily activities: working and studying (1.8 ± 1.2), social activities outside of the home (2.0 ± 1.3), ability to travel by car or bus for a duration greater than 30 min (2.1 ± 1.3), intimate relationships (2 ± 1.3), physical activities (2.2 ± 1.3), emotional health (1.8 ± 1.1), and frustration (2 ± 1.3). 

### 3.5. Opinions and Health-Seeking Behaviors

**Figure 3:**
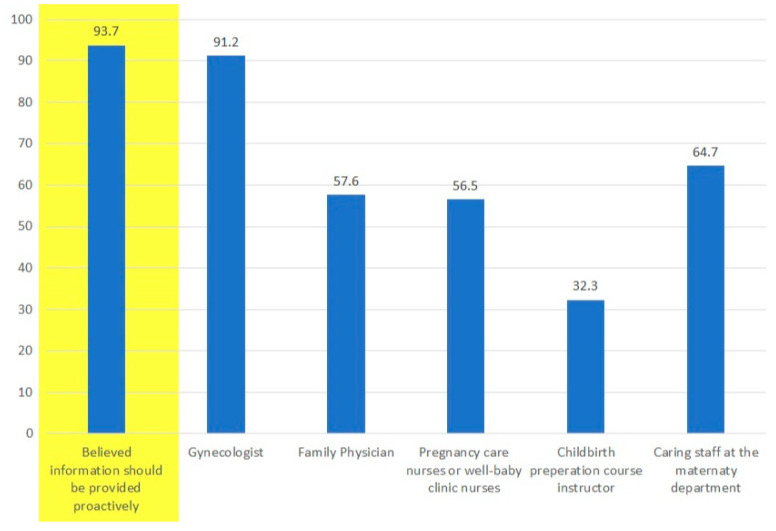
Participants answered that they wish to receive proactive information on PFD from healthcare personnel.

Most women in our study (93.7%) believed they should receive voluntary information on PFD symptoms from medical personnel (Figure 3). These included gynecologists (91.2%), family physicians (57.6%), the caring staff at the time of discharge from maternity wards (64.7%), pregnancy care nurses or well-baby clinic nurses (56.5%), and antenatal visits for childbirth preparation courses (32.3%). However, only 16.6% of women reported that a gynecologist or a family physician had ever initiated a conversation on these issues. 

Women also showed a low level of initiative in seeking information and treatment for PFD symptoms. Among women who experienced PFD symptoms, 70.8% (n = 371) never sought professional help, 32.3% approached a gynecologist on their own initiative, 22.8% approached a family doctor, 18.3% approached a pelvic floor physiotherapist, 8.9% approached a urologist, and 12.7% approached a nurse. In addition, 19.7% approached a fitness trainer or Pilates instructor on their initiative, and 42% shared this with a friend or acquaintance.

**Figure 4:**
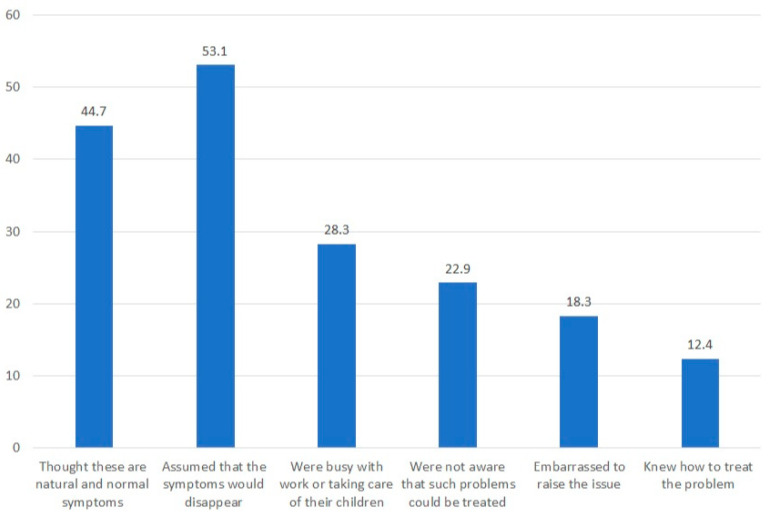
Reasons for avoiding healthcare in symptomatic women.

Regarding the reasons why most symptomatic women did not seek professional help, 53.1% assumed that the symptoms would disappear over time, 44.7% thought that these were natural and normal symptoms that every woman experiences after vaginal birth, 28.3% did not seek help because they were busy with work or taking care of their children, 22.9% were not aware that such problems could be treated, 18.3% were embarrassed to raise the issue, and 12.4% felt they knew on their own how to treat the problem with pelvic floor exercises (Figure 4).

Women who did seek medical care were offered numerous treatments: Kegel exercises for activating the pelvic floor muscles (46%), referral to physiotherapy for pelvic floor rehabilitation (29%), lifestyle changes such as reducing caffeine consumption and timed urination (17%), breathing exercises (15%), pharmacological treatment such as stool softeners and alpha-blockers (7%), and the use of supportive devices such as a pessary (1%).

**Figure 5:**
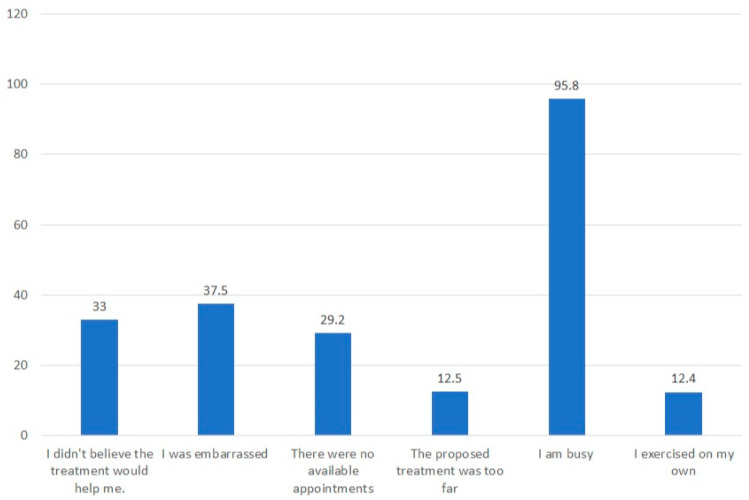
Reasons for low compliance with recommended treatment of PFD symptoms.

The degree of compliance with these treatments was inadequate, as only a small percentage of those who sought help followed through with all the recommended treatments (24.3%), while some completed only some of the treatments (20.3%) or planned to do so in the future (21.6%). Others did not complete the treatment and did not intend to (33.8%). The reasons for low compliance with recommended treatment were numerous and mainly stemmed from accessibility and awareness issues (Figure 5). Interestingly, 66% of those who reported no symptoms still sought treatment. This observation has several possible meanings, which will be discussed hereafter.

## 4. Discussion

### 4.1. Main Findings

In this study, we aimed to evaluate the prevalence of PFD among parous women in their fertile years using previously validated questionnaires and to characterize their approaches to treatment for these issues. To our knowledge, this study was the first in Israel to address women in their fertile years beyond the peripartum stage and to assess both symptoms and needs. 

The study results suggest that up to 95% of women experienced PFD symptoms, with 85% experiencing urinary-related symptoms (stress incontinence and/or overactive bladder). Moderate to severe symptoms were observed in 66.8% of women. Arab women, smokers, and overweight women were at a higher risk of suffering from PFD. 

Even though most symptomatic women (70.8%) did not proactively bring up their symptoms with any healthcare professional, almost all of them (93.7%) would have preferred to be approached and provided with information on the matter. Unfortunately, only 16.6% reported being actively approached by healthcare professionals. 

### 4.2. Interpretation

The prevalence of the symptoms observed in this study exceeds what is known in most existing literature [2,4,5,10,11,12,13,14,16,17], in which most studies have estimated up to 60% prevalence. This difference may be attributed to volunteer bias in the women who chose to answer the questionnaire. This is a known problem in any questionnaire study and is more predominant when discussing a sensitive subject such as PFD. This is even more true when answering a long and thorough questionnaire, as was used in our study. 

Another possible explanation is the gap between symptoms and bothersome symptoms. Some women were found to be symptomatic, but with no effect on their quality of life whatsoever and no wish for treatment, indicating that perhaps the USIQ and PFDI questionnaires were a little over-sensitive in our study. This might suggest that they were either experiencing mild levels of dysfunction, as evidenced by lower scores on the questionnaire, or they were adopting behaviors to cope with their symptoms. To diminish this effect, we chose to present women with moderate to severe symptoms separately—i.e., women with moderately to considerably bothersome symptoms. Even this restrictive analysis showed PFD to affect two-thirds of women in the study. Therefore, even if numerical accuracy has yet to be attained, it can be concluded that there is a considerable underestimation and underdiagnosis of these symptoms. 

It should be noted that sources for comparison on this subject are even more limited than those for PFD in general, as the medical literature tends to focus on these issues mainly in the peripartum or menopausal periods.

Regarding risk factors, obesity and smoking are known risk factors and were found in this study as well. It was interesting to find that Arab Israeli women were at greater risk of PFD, regardless of the type of delivery (assisted or not), the age, and the number of children they had. This specific finding should be further researched. 

One of the most important findings in our study was the discrepancy between women’s desire to be asked and offered voluntary information about PFD from physicians and nurses (93.7%) and the low rate of it happening (16.6%). We may assume that physicians and nurses avoid the subject due to its sensitive nature or that they assume that women will raise the issue if they find it bothersome. This should be further studied. These findings clearly show that most women would like to be asked about it, and the reasons for them not seeking treatment are mainly lack of knowledge and inaccessibility, and are not due to embarrassment. These are all solvable problems. 

A total of 66% of the women who reported no symptoms still stated that they sought treatment for PFD. This may be explained by the tendency to diminish symptom severity in self-reporting symptom scoring or by pursuing preventive treatments following parity.

Interestingly, when women did seek help for their symptoms, they tended to have low compliance with recommended methods. Only 24.3% of those seeking assistance completed all the recommended treatments. For the most part, the women were too busy to adhere, but other reasons included a lack of faith in the treatment, embarrassment, low availability, and self-management. These findings suggest that perhaps treating strategies in Israel may not correspond to a busy mother’s life, and perhaps new and more available technologies, including remote treatment and self-care devices, should be implemented. 

### 4.3. Limitations of the Study

This study has several limitations. First, a selection bias is probable. Women who chose to answer the questionnaire may suffer more or be more aware of PFD. Second, the response rate was only 44%, which may be improved using different approach models (telephone interviews, interviews in the clinic, etc.). Additional population biases stem from the research method and questionnaire distribution (via e-mail), including higher education and socioeconomic status, language, and technological literacy. This, again, can cause a selection bias. 

This study only represents parous women. However, it is essential to explore nulliparous women in future studies to represent their PFD. To fully comprehend the scope of PFD in young women, it is necessary to conduct research that involves a control group consisting of women from the same age group who have never undergone vaginal delivery. This is another population that suffers from misrepresentation in the literature. 

## 5. Conclusions

This study aimed to investigate the prevalence of female pelvic floor dysfunction in Israeli women who experienced vaginal delivery and are in their reproductive years, as well as to understand their attitudes and health-seeking behavior towards this problem. The topic is not novel. However, it is relevant in the field of pelvic floor dysfunction and addresses a specific gap in the field. Compared to the other published material, it adds further evidence to show the importance of raising awareness of the different therapeutic solutions to PFD symptoms and designing more available services for this common problem.

To date, unfortunately, there is still a cultural limit on addressing the problem of pelvic floor dysfunction with a gynecologist. In fact, to date, there is still the belief that these problems are inevitable and lack solutions. This leads women to accept this problem, with an enormous decline in their quality of life, without looking for possible solutions. It is the doctor’s duty to educate women to be aware of these problems, which, although they appear, can be treated with medical and surgical rehabilitation therapy. The possibility of having various therapeutic opportunities based on the individual patient allows the woman to access an individualized therapeutic plan. 

We anticipate that heightened awareness of this issue will eventually reach patients in diverse clinical settings, including doctor visits in various disciplines such as family medicine, gynecology, and urology, as well as during childbirth and parenting education courses, in nurses’ clinics, and upon discharge from maternity departments. Beyond discussing the matter in medical appointments, other effective health interventions could involve addressing the problem through pregnancy tracking apps or children’s developmental apps; distributing patient information leaflets, questionnaires, and signs; or providing specific pelvic floor examinations. Utilizing social media platforms or podcasts could also be effective in spreading awareness.

To tackle the accessibility challenges of care and treatment, it would be beneficial to establish standardized treatment plans, including remote pelvic floor physiotherapy and self-treatment solutions using readily available home devices. Subsidizing biofeedback tools for self-treatment could alleviate some of the significant accessibility barriers identified in this study.

This study highlights a significant yet often overlooked issue with a substantial public health burden. Various measures can be implemented to improve awareness, accessibility, and care in this regard. We believe that paying attention to patients’ needs could contribute to designing better solutions and promoting a healthier life for them.

## Figures and Tables

**Table 1:** Characteristics of women with and without at least one moderate to severe pelvic floor complaint.

	Women without Any Moderate to Severe Pelvic Floor Complaints n = 174 (33.2%)	Women with at Least One Moderate to Severe Pelvic Floor Complaint n = 350 (66.8%)	*p* Value
	n (%)	n (%)	
Age			0.173
18–24	11 (6.3)	29 (8.3)	
25–29	20 (11.5)	53 (15.1)	
30–34	50 (28.7)	83 (23.7)	
35–39	36 (20.7)	48 (13.7)	
40–44	31 (17.8)	77 (22)	
45–50	26 (14.9)	60 (17.1)	
Age, mean ± SD	35.9 ± 7.1	35.9 ± 7.9	0.938
Only vaginal deliveries	138 (79.3)	273 (78)	0.737
Number of vaginal deliveries, mean ± SD	2.24 ± 1.5	2.24 ± 1.4	0.881
Duration since last delivery			0.231
<2 months	11 (6.3)	16 (4.6)	
2–12 months	21 (12.1)	68 (19.4)	
1–5 years	74 (42.5)	134 (38.3)	
5–10 years	39 (22.4)	68 (19.4)	
>10 years	29 (16.7)	64 (18.3)	
Duration since last delivery (years), mean ± SD	5 ± 5	5.2 ± 5.3	0.861
Assisted vaginal birth (forceps/vacuum)	21 (12.1)	71 (20.3)	0.021
Perineal tear/episiotomy stitching	127 (73)	251 (71.7)	0.330
Hormonal therapy			0.767
OCP	37 (21.3)	69 (19.7)	
IUD	26 (14.9)	63 (18)	
Ethnicity			0.559
Jewish	143 (82.2)	279 (79.7)	
Arab	31 (17.8)	71 (0.23)	
Smoker	16 (9.2)	45 (12.9)	0.249
Academic education	103 (59.2)	221 (63.1)	0.658
Married	161 (92.5)	297 (84.9)	0.077
BMI	24.4 ± 4.1	25.8 ± 5.5	0.015

## Data Availability

The data supporting this study’s findings are available upon request from the corresponding author, T.F.-Y. The data are not publicly available due to ethical restrictions.

## Supplementary Materials

The following supporting information can be downloaded at: https://www.mdpi.com/article/10.3390/healthcare12030390/s1, Women’s Health Questionnaire.

## Abbreviations

CI	Confidence interval
OR	Odds ratio
PFD	Pelvic floor dysfunction
PFDI20	Pelvic Floor Disability Index 20
POP	Pelvic floor organ prolapse
TVT	Tension-free vaginal tape
USIQ	Urgency, Severity, and Impact Questionnaire
