# Supplementary material for: Pelvic Floor Dysfunction among Reproductive-Age Women in Israel: Prevalence and Attitudes—A Cross-Sectional Study
Source: Healthcare (Basel). 2024 Feb 2;12(3):390. doi: 10.3390/healthcare12030390 (PMC10855918; doi:10.3390/healthcare12030390)
Supplement: Supplementary file 1 [file healthcare-12-00390-s001.zip › healthcare-2773012-supplementary.pdf]

<u>Women's Health Questionnaire</u>

Pelvic floor weakness after childbirth affects almost two-thirds of women worldwide, causing symptoms such as pelvic organ prolapse, urinary incontinence, constipation, pain during sexual intercourse, and more. Despite the significant impact on their quality of life, many women with these symptoms do not seek help. Various reasons have been identified for this issue based on research conducted globally and in Israel. Our research aims to investigate the prevalence of pelvic floor dysfunction-related symptoms among women in Israel, explore the methods they use to cope with these issues, and identify the barriers preventing them from seeking help.

To participate in the study, we invite you to complete the Women's Health Questionnaire. Your responses to the questionnaire will remain confidential, will be aggregated with responses from all participants, and will be used for statistical purposes only.

1. Please indicate the age group to which you belong:
   - Up to 18
   - 18-24
   - 25-29
   - 30-34
   - 35-39
   - 40-44
   - 45-50
   - + 51

2. Have you ever had a vaginal birth, meaning a regular birth (not a C-section), and if so, how many vaginal births have you had?
   - I have not had any vaginal births/I have had a C-section birth(s) only.
   - I have had both vaginal and C-section births.
   - I have had vaginal births.

3. How many births have you had? _____
4. When was your last childbirth?

- Less than two months ago
- between two months and a year ago
- between two months and a year ago
- between 5-10 years ago
- More than 10 years ago

5. Below are pelvic floor related symptoms, please consider whether you have experienced them in the last 3 months and mark the appropriate answer:

0 - Not at all
1 - Yes and it doesn't bother me at all
2 - Yes - and it bothers me to a minor extent
3 - Yes - and it bothers me to a moderate extent
4 - Yes - and it bothers me to a great extent
5 - Yes - and it greatly bothers me

	0	1	2	3	4	5
A. Usually experience pressure in the lower abdomen						
B. Usually experience frequent urination?						
C. Usually experience urine leakage associated with a feeling of urgency, that is, a strong sensation of needing to go to the bathroom?						
D. Usually experience urine leakage related to coughing, sneezing, or laughing?						
E. Usually experience small amounts of urine leakage (that is, drops)?						
F. Usually experience difficulty emptying your bladder?						

G. Usually experience a feeling of incomplete bladder emptying?						

6. Below are the symptoms related to pelvic floor, please think about the symptoms you had or didn't have during the last 3 months and mark the appropriate answer:

0 - Not at all
1 - Yes and it doesn't bother me at all
2 - Yes - and it bothers me to a minor extent
3 - Yes - and it bothers me to a moderate extent
4 - Yes - and it bothers me to a great extent
5 - Yes - and it greatly bothers me

	0	1	2	3	4	5
A. Ever have to push up on a bulge in the vaginal area with your fingers to start or complete urination?						
B. Usually experience *pain* or *discomfort* in the lower abdomen or genital region?						
C. Usually experience heaviness or dullness in the pelvic area?						
D. Usually have a bulge or something falling out that you can see or feel in your vaginal area?						
E. Feel you have not completely emptied your bowels at the end of a bowel movement?						
F. Feel you need to strain too hard to have a bowel movement?						

G. Ever have to push on the vagina or around the rectum to have or complete a bowel movement?						

7. The following are symptoms related to pelvic floor, please think about the symptoms you have or haven't experienced in the past 3 months and select the appropriate answer:

0 - Not at all
1 - Yes and it doesn't bother me at all
2 - Yes - and it bothers me to a minor extent
3 - Yes - and it bothers me to a moderate extent
4 - Yes - and it bothers me to a great extent
5 - Yes - and it greatly bothers me

	0	1	2	3	4	5
A. Usually lose stool beyond your control if your stool is well formed?						
B. Usually lose stool beyond your control if your stool is loose?						
C. Usually lose gas from the rectum beyond your control?						
D. Usually have pain when you pass your stool?						
E. Experience a strong sense of urgency and have to rush to the bathroom to have a bowel movement?						

F. Does part of your bowel ever pass through the rectum and bulge outside during or after a bowel movement?						

The following questions related to urinary urgency (urgency refers to a sudden and strong urge to urinate, that is difficult to delay, as there is a concern of urine leakage):

8. During the past month, have you felt urgency (a strong and immediate need) to urinate?
   - Yes
   - No

9. During the last month, what proportion of your urinations had urgency associated with them?
   - None or almost none of the urinations
   - Some of the urinations
   - About half of the urinations
   - Most of the urinations
   - All or almost all of the urinations
   - Don’t know

10. When you have urgency, is it typically:
    - Extremely mild
    - Mild
    - Moderate
    - Severe
    - Extremely severe
    - Don't know

11. How long can you wait to urinate once you have urgency? Mark the answer that is most suitable for you.

- Half an hour or more
- Less than half an hour, but more than 5 minutes
- Less than 5 minutes, but more than a minute
- Less than 30 seconds
- I cannot hold it
- Don't know

12. During the last month, how much has urinary urgency bothered you?
    - None
    - Slight
    - Moderate
    - Considerable
    - Extensive
    - Don’t know

13. In an average 24 hour period how much of the time is urinary urgency present?
    - Never
    - Rarely
    - Sometimes
    - Usually
    - Always
    - Don’t know

14. How much does urinary urgency affects your:

0 - Not at all
1 - Somewhat
2 - Moderately
3 - Quite A Bit
4 - Always

	0	1	2	3	4	5
A. Ability to do household chores, do your work or schoolwork?						
B. Ability to do physical activities such as walking, swimming or other exercise?						
C. Ability to have an intimate relationship or sexual intercourse?						

D. Entertainment activities such as going to a movie or concert?						
E. Ability to travel by car or bus for a distance greater than 30 minutes away from home?						
F. Participating in social activities outside your home?						
G. Emotional health (nervousness, depression, etc)?<br>H. Feeling frustrated?						

Many women suffer from a multitude of symptoms that can be related to pelvic floor weakness after childbirth, such as pelvic organ prolapse, urinary incontinence triggered by exertion like coughing, sneezing, or the urge to urinate, constipation and fecal incontinence, pelvic pain, pain during sexual intercourse, and others.

15. Have you ever been asked by a Gynecologist or family doctor after giving birth if you suffer from such symptoms?
    - Yes
    - No
    - I don't remember.

16. Have you ever initiated contact with a medical professional regarding urinary incontinence or pelvic floor issues, such as the ones you were asked about in previous questions? If so, who did you contact and what was their gender? (If not, indicate so) (It is possible to mark more than one answer)

	Yes	No	I did not contact anyone
A. Gynecologist			
B. Family doctor			
C. Kegel exercise training (activating the pelvic floor muscles)			
D. Urologist			
E. Pelvic floor physiotherapist			
F. Lactation consultant/pregnancy support nurse.			
G. I consulted with friends			
H. Fitness trainer/Pilates instructor			
I. I didn't go to anyone			

17. What were the reasons for not seeking any medical attention regarding the symptoms you felt in your pelvic floor/lack of urinary control? (Multiple answers possible)
    - These are natural and normal symptoms that every woman experiences after childbirth.
    - I assumed that the symptoms would go away with time.
    - I was too embarrassed to bring up the issue with medical professionals.
    - I was not aware that such problems could be treated.
    - I do not believe that these problems can be effectively treated.
    - I am busy with work and/or taking care of my children and did not have the time to address this issue.
    - I was afraid that surgery would be suggested.
    - I already knew how to treat the problem through pelvic floor exercises.
    - I did not feel that my condition was severe enough to justify medical treatment.

- Other: ___________

18. What treatment was proposed to you? (Multiple answers possible)
    - Lifestyle changes - for example, reducing drinking, reducing caffeine intake.
    - Breathing exercises, guidance for Kegel exercises (sphincter muscle activation), referral to physical therapy for pelvic floor rehabilitation.
    - Treatment with a pessary.
    - Medication therapy - stool softeners, medication to control urinary incontinence.
    - Surgical treatment.
    - No treatment was proposed to me.
    - Other: ___________

19. Have you undergone any of the proposed treatments for pelvic floor rehabilitation? (Choose one)
    - Yes, I underwent all the proposed treatments.
    - Yes, I underwent some of the proposed treatments.
    - No, I haven't undergone any treatments yet, but I am preparing to.
    - No, I haven't undergone any treatments, and I am not planning to.

20. What were your reasons for not undergoing the recommended treatment? (Multiple answers possible)
    - I didn't believe the treatment would help me.
    - I was embarrassed to seek treatment for such an intimate problem.
    - There were no available appointments with a doctor at a reasonable time for me.
    - The proposed treatment was too far from my place of residence.
    - I am busy with work and/or taking care of my children, and I didn't have the time to address it.
    - I exercised on my own and didn't need additional help.
    - Other - please specify.

21. Many women worldwide suffer from a range of symptoms related to weakened pelvic floor after childbirth. There are various affordable treatments available for these problems under health insurance schemes.

Do you think this is information that a medical practitioner should proactively provide to their patients?

- Yes
- No

22. If yes, who do you think should provide such information to patients? (Multiple answers possible)
    - Gynecologist
    - Family Physician
    - Pregnancy follow-up/ lactation consultant nurse
    - Childbirth preparation course instructor
    - Upon discharge from the maternity department
    - Other- please specify

23. Do you think this is information that should be provided in childbirth preparation courses and/or during hospital discharge for the mother?
    - Yes
    - No
24. How old are you (in years) ____

25. How many vaginal deliveries (from third trimester onwards, including stillbirth) have you had? ________

26. How many years ago was your last delivery? ____

27. What types of vaginal deliveries have you had?
    - At least one instrumental delivery (vacuum/forceps)
    - Only vaginal non-instrumental deliveries

28. Did you require vaginal stitches after any of your deliveries?
    - Yes
    - No

- Don’t know/ Don’t remember

29. Do to smoke?
    - Yes
    - No

30. What is your current weight in KG? _____

31. What is your current height - in Meters? _____

32. Are you taking any hormonal treatment? If so, what is it?
    - Birth control pills
    - Hormonal intrauterine device
    - Copper intra-uterine device
    - I do not take any hormonal treatment.
    - Vaginal ring (Nuvaring)
    - Other-please specify _______________
33. For how many years, in total, have you been taking the treatment hormonal treatment? ______

34. What do you identify as?
    - Israeli Jew.
    - Israeli Arab
    - Other- please specify.

35. (If marked Arab) - What religion do you identify with?
    - Muslim
    - Christian
    - Other- please specify.

36. (If marked Israeli Jew) What is your ethnic origin?
    - Native-born of Ashkenazi origin

- Native-born of Mizrahi origin
- Native-born (of both Ashkenazi and Mizrahi origin)
- I am not native-born.
- Other-please specify ________

37. What is your education level?
    - Elementary
    - High school
    - Some college
    - Academic (college degree or higher)

38. What is your Marital status?
    - Single
    - married/living in a partnership, or co-parenting.
    - A single-parent mother
    - Divorced
    - Widowed
39. The average monthly income per individual, gross, is 10,800 nis. Is your income:
    - Way below average
    - A little below average
    - Around average
    - A little over average
    - Way over average

40. Where do you live (city/town): ____________

41. Which is your residential district:
    - North
    - Haifa
    - Gush Dan
    - Center
    - Shfela

- Jerusalem
- South

We thank you for your cooperation!
